# Cost-equivalence and Pluralism in Publicly-funded Health-care Systems

**DOI:** 10.1007/s10728-016-0337-z

**Published:** 2017-01-06

**Authors:** Dominic Wilkinson, Julian Savulescu

**Affiliations:** 1 Oxford Uehiro Centre for Practical Ethics, Faculty of Philosophy, University of Oxford, Suite 8, Littlegate House, St Ebbes Street, Oxford, OX1 1PT UK; 2 John Radcliffe Hospital, Oxford, UK

**Keywords:** Resource allocation, Responsibility, Cost-effectiveness, Medical ethics, Autonomy, Professional responsibility

## Abstract

Clinical guidelines summarise available evidence on medical treatment, and provide recommendations about the most effective and cost-effective options for patients with a given condition. However, sometimes patients do not desire the best available treatment. Should doctors in a publicly-funded healthcare system ever provide sub-optimal medical treatment? On one view, it would be wrong to do so, since this would violate the ethical principle of beneficence, and predictably lead to harm for patients. It would also, potentially, be a misuse of finite health resources. In this paper, we argue in favour of permitting sub-optimal choices on the basis of value pluralism, uncertainty, patient autonomy and responsibility. There are diverse views about how to evaluate treatment options, and patients’ right to self-determination and taking responsibility for their own lives should be respected. We introduce the concept of cost-equivalence (CE), as a way of defining the boundaries of permissible pluralism in publicly-funded healthcare systems. As well as providing the most effective, available treatment for a given condition, publicly-funded healthcare systems should provide reasonable suboptimal medical treatments that are equivalent in cost to (or cheaper than) the optimal treatment. We identify four forms of cost-equivalence, and assess the implications of CE for decision-making. We evaluate and reject counterarguments to CE. Finally, we assess the relevance of CE for other treatment decisions including requests for potentially superior treatment.

## Introduction and Cases

What should health professionals do if patients request medical treatment that is contrary to the accepted standard? The professional may believe that the requested option would be harmful (it would not yield the best outcome for the patient), and wasteful—(given superior options it would not be the best use of limited resources). Consider the following hypothetical cases (Box 1):

**Box 1:** Requests for suboptimal treatment

Case A: drugs to reduce transfusion need
Jim is a 38 year old man who is scheduled to have an elective major operation. Jim is anxious about his forthcoming surgery. In particular, he is worried about the possible need for a blood transfusion. He would prefer not to be transfused even if he has significant blood loss during surgery. Jim has read that recombinant Erythropoietin and supplemental iron would reduce his chance of needing a transfusion [23]. However, Jim’s haemoglobin level is normal, and national guidance states clearly that medical practitioners should not offer Erythropoietin to patients who are not anaemic [15].
Case B: smoking cessation
Julia is a 40 year old woman who has smoked heavily for 20 years. She strongly desires to give up smoking, and has made many previous unsuccessful attempts to do so. Julia has gone to her GP to request a drug to help her give up smoking. The GP offers her a prescription for Varenicline, a nicotine partial agonist that has been shown to be effective. However, Julia has heard about a naturally derived medicine (Cytisine). She strongly prefers natural remedies, and is worried about side effects from Varenicline. Current evidence summaries acknowledge that Cytisine may be more effective than Varenicline, however, because of a lack of large trials [24] there is some uncertainty and therefore it is not recommended.
Case C: double embryo transfer
Jane and Peter are academics in their late thirties. They have been trying to conceive unsuccessfully for several years. They are requesting IVF, and have specifically asked for two embryos to be implanted. National guidelines and local policy strongly encourage single embryo transfer for women of Jane’s age because of the increased risk of multiple birth with two embryo transfer, with consequent increased maternal and fetal complications [20]. However, Jane is not concerned about the risk of multiple birth. She was a twin herself. She would like to have several children, and is concerned that if she implants only a single embryo she will have to pay for further IVF cycles (they have limited financial resources), and will need to take a longer break from her career.

In the cases above, should the patients receive the treatment they are requesting? One initial thought is that it might matter whether they are requesting treatment in a private or public healthcare system. Choice about treatment is often seen as a defining feature of private healthcare. Greater choice can be a major motivation for taking out private health insurance [25].

In contrast, publicly-funded healthcare systems (PHS) do not routinely offer choice around treatment. To ensure that patients receive consistent, high quality care, PHS often develop and disseminate clinical guidelines that assess available evidence and provide recommendations about optimal treatment (for example, the National Institute for Health Care Excellence (NICE) in the UK).

In this paper, we will focus our attention on PHS. Our discussion will draw on the example of the National Health Service (NHS) in the UK, since that is familiar to us, and provides useful, relevant, and accessible examples of national best practice clinical guidelines. However, the issues below are not restricted to one particular PHS. They apply, for example, to Medicaid patients in the US, many of whom may be able to access only treatments available through a managed care plan [41]. They are also relevant for private healthcare systems, since such systems frequently make stipulations about which treatments will be provided and which will not.

We concentrate on the boundaries of choice for competent adult patients. However, the basic principles of the analysis will still be relevant for at least a subset of paediatric treatment decisions.^1^

We restrict our discussion to conventional medical treatments that have been scientifically evaluated to have some medical benefit relative to no treatment and are prescribed or provided by registered health professionals. We will assume, for the purpose of simplicity, the framework used in many PHS to appraise treatments in terms of cost per quality-adjusted life year gained (Cost/QALY). It is not the purpose of this paper to assess or analyse different methods for evaluating the cost-effectiveness of treatments; the framework of cost-equivalence could be applied to other methods for assessing cost-effectiveness.

Finally, it is worth noting at this point what it might mean for a treatment to be ‘suboptimal’ (Table 1). Treatments can be inferior to other available options because they yield a smaller benefit, because they increase risks for the patient, because they are more costly, or because of relative lack of evidence.

**Table 1:** Different ways in which treatment might be suboptimal

Type of suboptimality	Example
Reduced magnitude of benefit	Smaller improvement in symptom scores
Reduced probability of benefit	Reduced probability of live birth after in vitro-fertilisation
Reduced duration of benefit	Reduced median survival with cancer treatment
Increased magnitude of harm	Risk of death rather than risk of stroke
Increased probability of harm	Increased probability of heart attack
Increased cost, but similar effectiveness	Drug to reduce post-operative anaemia is considerably more expensive than standard care, but doesn’t improve outcome (or does only by a very small amount)
Reduced evidence	Uncertainty about relative benefit/harm, or about costs

## Against Sub-optimal Treatment

There are two essential arguments in favour of health professionals (or health systems) providing only optimal medical treatment according to their best judgment.

First, health professionals have a duty to aid and not harm patients. The principles of beneficence and non-maleficence lie at the heart of evidence-based medicine. Doctors should critically and impartially assess evidence about medical treatments in order to determine which treatment is best for a given condition, or for a given group of patients [22].

The principles (or duties) of beneficence/non-maleficence do not mandate that physicians always do the best possible. The best treatment may be unaffordable, or may be very limited in availability. Rather, these principles require physicians to provide the best and least harmful *available* treatment option(s).

*The Optimal Treatment Principle* *: For a given condition, Publicly*-*funded Healthcare Systems should provide only the most effective treatment that is both available and affordable.* 2

Of course, the patient may decline this treatment. In such circumstances, the value of autonomy comes into conflict with that of beneficence. For a competent adult patient, such refusal should be respected. However, that does not negate the importance of determining, recommending and providing (if desired) the best available medical treatment. In Jim’s case, for example, best practice guidelines recommend that he be offered tranexamic acid (a drug to reduce blood loss during surgery) [15]. Intraoperative cell salvage (where blood lost during surgery is given back to the patient) might be considered [15]. However, a physician might well feel that Erythropoietin should not be provided because it would potentially impose risks on Jim, for little or no benefit.

Second, health care providers should provide optimal treatment because this represents the best use of limited medical resources. Publicly funded healthcare systems have a finite budget, and there are constraints on the resources available to treat patients. Providing optimal medical treatment represents an important way of securing the greatest health benefit possible from this limited resource. This includes attention to the cost of treatment, as well as to the benefit of providing that treatment. These can be combined formally in appraisal of the cost-effectiveness of treatment [31]. This also applies to private health insurance, since such bodies have to make decisions about allocating the finite financial resources gained from policy subscriptions. Traditionally, coverage decisions for insurers have been based on evidence of effectiveness (rather than cost-effectiveness), providing “medically necessary” therapies [17]. However, some have argued that private insurers also need to explicitly take account of the cost-effectiveness of treatments [17].

Cost-effectiveness is not the only priority for health systems. There may be reasons to depart from it (for example, because of a desire to provide equality of access to treatment, or priority to the worst off). Nevertheless, cost-effectiveness represents a centrally important principle for public (and private) healthcare systems.

Many publicly-funded healthcare systems apply a threshold to treatments. The Incremental Cost Effectiveness Ratio (ICER) represents the additional cost per additional quality-adjusted life year compared with the standard of care. Those treatments that provide sufficient incremental benefit (relative to their cost) are funded [31]. Conversely, treatments that exceed the ICER threshold are not provided. Countries vary in how they apply ICER thresholds, and the level at which they apply. Which treatments count as optimal will depend on the ICER threshold used and will depend on the available alternatives. As an example, the UK National Institute for Health and Care Excellence (NICE) does not usually recommend provision of treatments assessed as having an incremental cost of than £20-30,000/QALY [9, 13]. In Jim’s case, a relevant study (assessing the use of Erythropoietin for mildly anaemic patients prior to orthopaedic surgery) assessed it as costing an additional £1235 per patient, for a gain of 0.00006 QALYs [15]. This amounts to more than £21million/QALY.

## In Defense of Sub-optimal Treatment

There are strong reasons for physicians and PHS to endorse the Optimal Treatment Principle. So why, then, should they consider providing sub-optimal treatment?

One reason is given by *value pluralism*: within any (democratic) society there will be a plurality of value systems, and a diversity of views about how to live [44]. People’s views diverge about a range of fundamental questions, political, ethical and religious. This diversity appears to be inevitable and irresolvable. It is not possible to determine a single correct view or set of values. As a consequence, negotiation, tolerance and compromise are necessary. As suggested by Table 1 above, there are different ways in which treatments may be judged to be superior or inferior. Treatments will often be better in some ways, but worse in others. For example, Jane and Peter place greater value on the potential benefit of having two children (and twins) than on avoiding particular medical risks. Julia places greater value on receiving a naturally derived treatment, than on the greatest scientific evidence. Treatments that are most effective on average for a population will be not always be most effective in light of the goals and values of specific individuals within that population. Others in society may not share those values, but that does not make them wrong. Furthermore, allowing choice causes patients to take more responsibility for their health care decisions and increases participation in decision-making.

A second, related justification for permitting sub-optimal treatment is based on the value of patient autonomy. Even if people are mistaken in their factual beliefs about treatment, or in the values that they apply to treatment decisions, in general we think that competent patients’ decisions about medical treatment should be respected.

Autonomy is often understood as a negative right – giving competent patients the absolute freedom to refuse treatment that they do not desire, no matter how beneficial it might be. It does not represent a positive right to demand whatever medical treatment is desired [32]. However, a purely negative account of patient autonomy seems too thin. The value of autonomy lies in the importance of self-government and freedom to live according to one’s goals [45]. Decisions by health professionals and health systems that preclude options that are important to patients significantly constrain self-government. When patients can choose according to their values, they can take responsibility for their health care, and the results of it.

A third reason arises from *uncertainty* in determinations about optimal treatment. Scientific studies allow comparison between different potential treatment options. However, in many cases, there will be some significant uncertainty about these estimates. (For example, it may be unclear whether data obtained in the context of a randomized controlled trial can be extrapolated to a different setting or to the general population). Even if such scientific uncertainty were minimal, other types of uncertainty would remain. For example, evaluations of the impact of treatment on quality of life are typically based on ratings by the general public of the value of survival in different health states (described in terms of a combination of attributes) [6]. Yet there can be significant differences in estimates of QALY using different methods, or different populations; the precision of such estimations can obscure the underlying uncertainty about how to assess quality of life, and how to incorporate it into cost-effectiveness calculations. Uncertainty, in cost-effectiveness assessment can generate different views about which treatments would be best.

Value pluralism, uncertainty about evidence, and genuine respect for patient autonomy and responsibility suggest that physicians (and PHS) should be prepared to provide at least some sub-optimal treatment options. But where should the boundaries of those options be? Drawing on Mill, a liberal account of medical treatment would permit patients to choose medical options, *as long as they do not cause harm to others* [28].

### Cost-equivalence

In a closed PHS, one important way in which treatment choices by an individual could harm others is through consumption of limited resources. This also potentially applies in private healthcare systems, since excessive costs of treatment for one patient could be reflected in increased insurance premiums (or reduced coverage) for others.

Of course, costs for individual patients do not necessarily translate to harms to others [12]. In private systems, increased costs could lead to reduction in profits (with no change in coverage for others). In publicly-funded healthcare systems, increased costs could lead to a higher health budget being assigned, and reduced funds available for other (non-health) priorities or to increased taxation. However, in both systems, provision of more expensive options has the potential to lead to harm to other patients via reduced access to treatment. This gives rise to one principle that we could use to determine the permissibility of substitute treatment.

*The Cost* - *Equivalence Principle* *: As well as providing the most effective, available treatment for a given condition, Publicly*-*funded Healthcare Systems should provide reasonable suboptimal medical treatments that are equivalent in cost to (or cheaper than) the optimal treatment.*

We could apply this straightforwardly to treatment determinations. The idea of cost-equivalence is that patients who request substitute treatments receive the same (financial) support as they would have received if they had accepted the optimal treatment. Their decision to choose substitute treatment does not require any additional resources. It does not lead to other patients being denied treatment. This seems, prima facie, to be a fair allocation of resources. It is also, at least from the point of view of individual preferences *Pareto Superior*.^3^

Pure cost-equivalence (CE) (Table 2) focuses only on the cost of the requested substitute treatment. If we consider a set of hypothetical suboptimal treatments (Box 2), CE would support any treatments that are equal or lower in total cost than the optimal treatment.

**Table 2:** Different potential versions of cost-equivalence

Variants of cost-equivalence	
Pure cost equivalence (CE)	Where PHS is prepared to provide treatment A, provide reasonable substitute treatment B iff Cost_B_ is ≤Cost_A_
Cost-effectiveness equivalence (CEE)	Where PHS is prepared to provide treatment A, provide substitute treatment B iff Cost_B_ is ≤Cost_A_ and Cost_B_/QALY_B_ ≤ Cost_A_/QALY_A_*
Cost-effectiveness threshold equivalence (CETE)	Where PHS is prepared to provide treatment A, provide substitute treatment B iff Cost_B_ is ≤Cost_A_ and CostB/QALY_B_ is ≤Cost Effectiveness Threshold**
Refusal cost-equivalence (RCE)	Where the cost of refusing treatment is >cost of optimal treatment A, and a PHS is prepared to absorb the costs of refusing treatment A, provide substitute treatment B iff Cost_B_ is ≤Cost_refusal_

* Substitute treatments can be more cost effective but still sub-optimal if they are less effective overall (and cheaper), or where there is uncertainty about effectiveness eg Cytisine

** The reason for restricting CEE and CETE to treatments that are less expensive than the optimal treatment is because this ensures no negative impact on overall health budgets, and Pareto optimality. Permitting requests for substitute treatment that are more expensive than the optimal treatment (albeit within the ICER threshold) would lead to increased health expenditure

**Box 2:** Hypothetical example of different novel cancer therapies (drug names are fictitious). Which should be provided in a Publicly-funded Healthcare System? For the purposes of this example, it is not necessary to specify the standard care. The new anti-cancer drugs will be prescribed in addition to standard care (not replacing standard care)

*A public healthcare system is evaluating whether to fund new life extending cancer treatments. Various supplementary treatments have been assessed in comparison with the current standard of care*	
*Axemab costs an additional £10,000 per treatment, and on average extends life by an additional 1 quality*-*adjusted life*-*year (QALY)*	
*Boximab costs the same amount, but extends life by only 0.5QALY on average*	
Cliximab is more expensive, but also more effective than treatment A. It costs £20,000 per treatment, but extends life for 1.5 QALYs*	
*Daxamab costs £10,000 per treatment, but extends life for only one week*	

* Cliximab is the most effective treatment, and falls within the incremental cost-effectiveness threshold for the UK. Axemab, Boximab and Daxamab may be cost-equivalent (depending on the version of cost-equivalence used). See Table 3

One potential concern about pure cost-equivalence is that it might require a health system to provide a highly expensive treatment for very limited benefit (e.g. Daxamab, Box 2). This seems counterintuitive. A second concern is that, contrary to the claim above, such a policy *could* have implications for other patients. A policy of pure cost-equivalence would lead to some patients being deprived of more beneficial treatment, wherever it would increase the uptake of medical treatment. For example, the annual budgetary impact of introducing Varenicline in the UK NHS was estimated at £7 million per year by 2011 (after its introduction in 2007) [43]. This was based on models assuming that 25% of eligible smokers would take prescription treatment. However, we might imagine that if a PHS decides to provide Cytisine as a cost-equivalent smoking cessation aid (case 2), a higher proportion of smokers would potentially take up prescriptions (e.g. 30%). In that situation, pure cost-equivalence would potentially lead to an increase in the total budgetary impact. In a closed publicly-funded healthcare system it would potentially affect the availability of treatment for others. This would provide a non-paternalistic objection to CE in some cases.

#### Refusal Cost-equivalence

In some circumstances optimal treatments will be cost-*saving* relative to not providing the treatment [37]. For example, influenza vaccination in older patients has been estimated to save $17 per person vaccinated [27]. Does that mean that sub-optimal treatments cannot be cost-equivalent? There are two alternatives. The first alternative would be to accept that in such circumstances sub-optimal treatments are not cost-equivalent. If patients wish to receive them, they could pay the full cost either within the PHS, or outside it (obtained privately). The second alternative is more radical. It depends on whether it is permissible for patients to refuse treatment, and whether they would be required to pay extra for any additional healthcare expenses that this incurred. For example, imagine that Jim’s reason for not wanting to have a blood transfusion were because he is a Jehovah’s Witness [37]. If he sustains severe blood loss during his surgery (and declines transfusion), he may end up having a more complicated and prolonged post-operative course [3].^4^ Should he be required to pay for that additional expense? It is beyond the scope of this paper to address the debate on individual responsibility and eligibility for publicly funded healthcare [7]. However, if we think that it acceptable for the PHS to absorb the costs of *refusal* of treatment, it would then seem unfair to impose additional costs for suboptimal treatment. This gives rise to an additional cost-equivalence principle—refusal cost-equivalence (RCE, Table 2). RCE could be combined with other cost-equivalence principles.

### Reasonableness

Should a PHS provide a very ineffective suboptimal treatment? If we are serious about value pluralism, and autonomy perhaps societies should be prepared to respect a patient’s decision about treatment, even where this diverges substantially from the choices that others would make. Such choices would still potentially be Pareto improvements. But one question is whether Daxamab represents a *reasonable* substitute treatment.

In the above account we proposed that PHS should provide *reasonable* cost-equivalent treatments. Defining this element is challenging, and may not be possible without begging the question. For many treatments there will be different views about whether or not they are reasonable.

Here are two possible ways of defining reasonable suboptimal treatment.

#### Pragmatic Account

*A* *Reasonable suboptimal treatment* *is one that: i. has been scientifically appraised, and there is reliable evidence about both its effectiveness and cost and ii. evidence suggests (though may not be conclusive) that it is more beneficial (relative to harms) than no treatment and iii. at least some qualified medical practitioners are prepared to provide the treatment.*

The first condition above is necessary for cost-equivalence to be assessed. When PHS considers the cost of treatments, they must take into account the up-front costs of the treatment and the long-term costs of illness and complications arising subsequent to providing the treatment. Sub-optimal substitutes may be cost-equivalent in the short term, but could be more expensive in the long-term if they lead to a greater burden of illness or to more medical complications. If there is evidence of greater long-term costs associated with a sub-optimal substitute those should be included into an assessment of whether it is cost-equivalent. Conversely, if there were no scientific evidence about the effects or costs of a treatment it will be impossible to assess whether providing it within a PHS would be permissible.

Why require even a minimal level of evidence of benefit? We might justify this in terms of the reasons for providing care within a PHS at all. Treatments are provided because they potentially contribute to a patient’s health and wellbeing. If there is no scientific evidence of health benefit, on this account there is no positive reason to provide it. (Although we have focused discussion in this paper on conventional medical treatment, this requirement would potentially exclude many complementary or alternative medicines).

On the first two conditions, it may be reasonable to provide the hypothetical cancer treatment Daxamab (Box 2)—but there would also need to be a professional willing to prescribe it. The fact that professionals will provide it does not provide a guarantee of reasonableness (the professionals might hold unreasonable views), however, the *absence* of professionals willing to provide a given treatment might be thought to provide fairly reliable evidence that this is not a reasonable option. Even if it were reasonable, if there are no health professionals willing to provide the treatment, it suggests that it is not a workable option within the PHS.

#### Reasonable Cost-effective Substitutes

An alternative approach to determine reasonableness would draw on accepted principles of determining and comparing cost-effectiveness of interventions. As a minimum, an intervention that has equivalent effect (relative to cost) to the optimal treatment, would be clearly reasonable (cost-effectiveness equivalence, CEE, Table 2). A policy of CEE would avoid the problem noted above that in some situations pure cost equivalence could impact the availability of treatment for other patients. It would allow patients to choose suboptimal substitute treatments, but it would prevent them from choosing less *cost*-*effective* treatments. This would have the advantage of ensuring that the health system allocated resources consistently and secured the greatest health benefit for the money invested in healthcare.

Yet, one concern with CEE is that it may be unduly restrictive. If an incremental cost-effectiveness ratio (ICER) threshold is used to guide the provision of treatment, we might expect that non-funded treatments are *at or above* the ICER threshold. In that case, it would be Pareto superior to allow sub-optimal choices as long as they do not exceed this level. This would yield a policy of cost-effectiveness threshold equivalence (CETE, Table 2).^5^

Table 3 summarises the implications of these different policies for our hypothetical new drugs.

**Table 3:** Choosing suboptimal treatments. The implications of 4 different policies on provision of hypothetical drugs

	Axemab	Boxemab	Cliximab	Daxamab
Cost (pounds)	10,000	10,000	**20,000**	10,000
Effect (QALY benefit)	1	0.5	**1.5**	0.02
Cost/QALY (pounds)*	10,000	20,000	**13,333**	500,000
Optimal treatment			**✓**	
CE	**✓**	**✓**	**✓**	**✓**
CEE	**✓**		**✓**	
CETE	**✓**	**✓**	**✓**	

Optimal treatment (highlighted in bold)—Treatment that secures the greatest absolute health benefit is defined as optimal (as long as it lies within the ICER threshold)

*CE* Pure cost equivalence; (any substitute that is equally or less costly than optimal treatment will be CE)

*CEE* Cost-effectiveness equivalence (any substitute that has a equal or lower Cost/QALY than the optimal treatment will be CEE)

*CETE* Cost-effectiveness Threshold Equivalence (any substitute that is equal or less costly than the optimal treatment and falls within the ICER threshold will be CETE)

* Incremental cost-effectiveness—compared with standard treatment

**✓** Indicates that the drug would be provided

#### Applying Reasonableness

Our accounts of reasonableness should be seen as complementary rather than in competition. The pragmatic account would be a valuable rule of thumb, but the notion of reasonable cost-effective substitutes might be used to help professionals decide whether or not they should offer a suboptimal treatment.

Note that in our account of cost-equivalence, we focus on the reasonableness of treatment substitutes, rather than the reasonableness of the *request*. Our focus on reasonableness of treatment is deliberate and has two advantages. First, it is considerably easier and less controversial to determine reasonable medical treatments (at least on the definition that we have provided) than to distinguish between reasonable and unreasonable justifications. For example, would it make Jim’s request reasonable if he were concerned about the risk of transmission of a novel (not yet discovered) blood-borne infection through transfusion? Would his request be reasonable if it were based on a religious doctrine? Some might regard these alternative justifications as reasonable, while others would not. Our account sidesteps those questions. Second, determining the reasonableness of treatment is more reliable, and less malleable than determining the reasonableness of a request. Third, determining the reasonableness of specific treatments potentially avoids problems of inconsistency between clinicians in determining the reasonableness of specific requests. If a PHS determines that Erythropoietin is a reasonable (if sub-optimal) option pre-operatively for patients with mild/no anaemia, then there is less risk that whether Jim’s request will be granted will depend on whether or not he finds a sympathetic practitioner.

### Applying Cost-equivalence

Whichever cost-equivalence policy is adopted, there are several different ways in which cost-equivalence could impact on decisions about treatment.

#### Binary Cost-equivalence

The most obvious way of applying cost-equivalence would be in a simple binary fashion: suboptimal treatment would be provided if it is below the Cost Equivalence threshold. If the suboptimal treatment were above the threshold, it would not be provided.

#### Cost-equivalence Through Altered Duration/Dosage

However, it might still be possible to achieve cost-equivalence for more expensive suboptimal treatments by providing them in a reduced quantity. For example, one randomized trial of treatments for lower back pain compared exercise prescriptions with massage or with various durations of lessons in Alexander technique [18]. In the trial, 6 sessions of massage was more expensive, but also less effective than 6 lessons in the Alexander technique. On the basis of evidence like this, a PHS might regard massage as suboptimal, and decide not to publicly fund it. But if a patient with chronic back pain strongly preferred massage over Alexander technique lessons, one cost-equivalent alternative would be to fund a shorter course of massage (e.g. 4 or 5 sessions of massage).

For suboptimal treatments that are within the cost-equivalence threshold, one possibility is that cost-equivalence may allow an *increased* dose or duration of cheaper treatment. For example, recall the case of Julia, who is unable to access treatment with Cytisine to help stop smoking. Currently, the cost of a standard course of Varenicline for smoking cessation is £163.80, while the cost of Cytisine is only £16.79 [24]. On a pure CE policy, it would appear reasonable to provide a considerably longer (even ninefold) course of Cytisine in place of Varenicline.

#### Cost-equivalence Through Reduced Price

Finally, for suboptimal treatments that are not currently cost-equivalent it may be possible to achieve cost-equivalence through a price reduction.

There are two ways of achieving such a price reduction. It might occur through negotiation. For example, a PHS might negotiate with a pharmaceutical company to reduce the unit price of the substitute drug—until it reached the cost-equivalent price. Alternatively, PHS (or insurers) might agree to subsidise part of the cost of a treatment—up to the relevant cost-equivalence point, with the patient paying a top-up amount or co-payment. Table 4 illustrates the different levels of top-up (or negotiated discount) required for our hypothetical drugs, on the basis of different policies.

**Table 4:** Co-payment for suboptimal treatments. The implications of 4 different policies on provision of hypothetical drugs

	Axemab	Boxemab	Cliximab	Daxamab
Cost (pounds)	10,000	10,000	**20,000**	10,000
Effect	1	0.5	**1.5**	0.02
Cost/QALY (pounds)*	10,000	20,000	**13,333**	500,000
Optimal treatment			**✓**	
CE	**✓**	**✓**	**✓**	**✓**
CEE	**✓**	**£3333	**✓**	**£9733
CETE	**✓**	**✓**	**✓**	**£9400

Optimal treatment is highlighted in bold

* Incremental cost-effectiveness—compared with standard treatment

**✓** Indicates that the drug would be provided without co-payment

** Numerical values indicate the patient co-payment (or price reduction) required to render the suboptimal treatment cost-equivalent

## Counterarguments to Cost-equivalence

### Objectivism and Strength of Claims

Cost equivalence respects the subjective preferences of patients for suboptimal treatment. However, one reason to resist cost-equivalence might be a belief that the strength of a claim for medical treatment (and the priority that should be given to it) is ultimately based upon its objective rather than its subjective value. For example, Scanlon argues that “[t]he strength of a stranger’s claim on us for aid in the fulfillment of some interest depends upon what that interest is and need not be proportional to the importance he attaches to it.” [38] Some may feel that patients’ claims to suboptimal treatment are weaker or less urgent than the claims of other patients for (objectively) optimal treatment. However, the arguments advanced in favour of cost equivalence are independent of whether the value or benefit of treatment is objectively or subjectively conceived. We are not endorsing subjectivism or relativism about health benefits. On the contrary, we accept that PHS are justified in evaluating treatments in terms of objective benefits.

If we take Scanlon’s argument to mean that only claims to treatment that are linked to objective benefit are important, some suboptimal treatments (at least those that are cost-effectiveness-equivalent) should still be provided (for example, in reduced dose or for reduced duration see “Reasonable Cost-effective Substitutes”). However, we have argued that uncertainty about the benefit of different treatments, the value of autonomy and respect for plural values gives us strong reason to accept and respect claims for cost-equivalent but objectively suboptimal treatment.

### Complicity in Harmful Choices

One concern about providing sub-optimal treatments is that this would encourage patients to make unwise choices or make the physician morally complicit in them. For example, Jane and Peter’s obstetrician may feel strongly that it would be wrong to implant two embryos.

We have earlier suggested that restricting patients’ options for their own benefit is unreasonably paternalistic. However, in the context of exploitative contracts, Shiffrin has argued that refusals that are motivated by concern to avoid personal complicity can be justified without being a form of paternalism [39]. On that basis, perhaps Jane and Peter’s obstetrician would be justified in refusing to implant two embryos because she judges it to be incompatible with her professional role?

Yet, *providing* a treatment option, after counseling and full information provision is not the same thing as *supporting* a treatment option. If it were an option for Jane and Peter to have double embryo transfer, it would be entirely reasonable (and compatible with their professional role) for a physician who believes that the decision is unwise to counsel against such a choice [35, 36]. Moreover, it is not clear that suboptimal (and cost-equivalent) treatment options are immoral and therefore able to generate a justifiable sense of complicity. If it is wrong for physicians or PHS to provide a suboptimal medical treatment (that is cost-equivalent and hence will not harm others) that can only be because of concern for the wellbeing of the patient. It is hard to see how that is not a form of paternalism.

Indeed, there are three ways in which our proposal might potentially lead to better, less harmful choices. The first is that disclosure of all reasonable options respects patients’ autonomy fully, and is valuable even if patients subsequently choose the recommended (optimal) treatment. By giving reasonable options, it encourages patients to take responsibility for their treatment, rather than acquiescing to the only option offered. Second, failure to provide substitutes may encourage an even worse choice—of forgoing treatment. Third, failing to provide options may lead patients to seek those options from other providers—where it is possible that they will not receive the best advice about treatment. For example, Jane and Peter may choose to travel overseas to access IVF, and end up having double embryo transfer or even four or five embryo transfer because of unregulated treatment and poor counseling [11].

### Harmful Externalities

We have argued that suboptimal cost-equivalent treatments should be provided since they will not cause harm to others. However, suboptimal treatment could cause harm in other ways. For example, a less effective form of a vaccine may be considerably less effective at generating herd immunity, or a less effective treatment for HIV might lead to more transmission of the virus to third parties.

One response to these concerns would be to take into account the costs of externalities in assessing whether or not a treatment is cost-equivalent. A flu vaccine that is much less effective may not be cost-equivalent if it leads to more cases of symptomatic flu in contacts of the patient.

However, if a less effective form of treatment causes harmful externalities of this sort it is highly likely that treatment refusal would cause even greater harm. That should lead us to ask whether it is permissible to refuse treatment. If it is not permissible to refuse treatment, it may not be permissible to receive suboptimal treatment either. It refusal is allowed, the relevant question may be whether the suboptimal treatment is refusal-cost-equivalent.

### Against Co-payment

One objection to cost-equivalence would be on the basis of ethical concerns about equality within a Publicly-funded Healthcare System [40, 46]. Some argue that it is unjust for patients with greater financial resources to be able to access options within a PHS that are not available to all [5].

However, this concern would not create a distinct objection to cost-equivalence in countries that already permit co-payments for some medical treatments in the PHS (USA, Canada, Australia, many others) [34], nor for private health care, where co-payments are commonly applied. This objection would also not prevent the use of cost-equivalence without co-payment (for example, where the suboptimal treatment is less expensive e.g. Cytisine, or by reducing dose/duration of treatment funded).

It is also not clear that the standard objections to co-payment provide convincing ethical arguments against cost-equivalence for sub-optimal treatment (Table 6 in Appendix 1). In particular, co-payment for treatments that are judged to be suboptimal, cannot increase inequality in health outcomes. Indeed, since wealthier patients may be able to choose inferior treatments, and thus secure for themselves worse health, *a contrario*, equality provides an argument in favour of cost-equivalence through co-payment!

### Supplementary Rather than Substitute Treatment

The approach that we have described would not apply to cases where patients desire sub-optimal treatments *in addition to* the most effective available treatment. For example, Julia might request Cytisine to help her stop smoking after previously having tried Varenicline. Where a suboptimal treatment is requested in addition to standard treatment, it will not be cost-equivalent. In that case, patients would be required to pay all of the cost of the supplementary treatment.

Supplementary treatment could be converted into substitute treatment. Patients might choose to forego certain existing treatment options in order to gain access to their desired suboptimal treatment. For example, Jim might refuse transfusion in order to gain access to Erythropoietin. This might lead to two qualms. It might lead some patients to manipulate the cost-equivalence system by declining future treatments, but later revoking their choice if they needed to. Alternatively, patients like Jim might genuinely change their minds about therapy once they require it, and physicians may feel compelled to give the treatment.

In situations where patients are contemplating foregoing beneficial treatment, as is presently the case, physicians should strongly discourage such choices, but ultimately should respect the patient’s autonomous decision to do so. If Jim declines transfusion, but later has become severely anaemic, the physician should encourage Jim to accept a transfusion. At that point, there would be the option of retrospectively charging a co-payment for the Erythropoietin, or of waiving the co-payment on compassionate grounds. If a large number of patients end up changing their minds about treatment, that could be incorporated into the costs of substitute treatment—that may render the alternative non-cost-equivalent.

## Further Applications of Cost-equivalence

### Cost-equivalence Between Patient Groups

Some *treatments* are less beneficial than others, and an efficient PHS might choose only to provide the most effective treatments (call this Intrapersonal Optimal Treatment). We have argued that Cost equivalence would allow patients to access desired suboptimal treatments without thereby harming any other patients.

However, there is an alternative possible application of cost-equivalence in a setting where sub-optimal treatments are sometimes not provided in a PHS. Given scarce public health resources, some *patients* would have less benefit from treatment than other patients. PHS may decide on the basis of cost-effectiveness to allocate treatment only to subgroups of patients with a higher likelihood of benefit (we could call this *Interpersonal* Optimal Treatment). For example, the chance of live birth with in vitro fertilization varies with maternal age. In 2010 in the UK, for women aged under 35, 32% of IVF cycles (with own eggs) resulted in live birth, compared with only 14% for women aged 40-42, and 2% for women aged over 45 [19]. A number of countries restrict access to IVF based on maternal age [14]. Although there are a variety of different possible rationales for such a policy [29], one potential ethical justification is on the basis that the lower effectiveness of IVF for older women justifies giving them a lower priority for treatment.

Cost-equivalence might be used to increase access to desired treatment for patients in worse prognostic groups. That could be in three ways:

First, on Pure Cost-Equivalence grounds, equal access to treatment might be provided regardless of prognosis. That would offer a more egalitarian approach to allocation, though with the implication that less benefit overall would result from providing treatment. It might mean providing publicly funded IVF in situations with very low chance of success.

Second, as noted above, Cost-Effectiveness Equivalence can be achieved by adjustment of the duration or dose of treatment. One possibility, then, would be to provide lower duration or quantity of treatment for patients with a worse prognostic group. Indeed, the UK policy on public access to IVF appears to do just this. UK national guidance recommends a maximum of three cycles of IVF for women aged less than 40, while offering one cycle to women aged 40–42 and none to women > 42.

Third, it would be possible to achieve Cost-Effectiveness Equivalence for patients with worse prognosis by reduction in the price of treatment. This could be achieved through negotiation^6^ or co-payment. Co-payment as a means for patients (in worse prognostic groups) to access treatment might be thought to raise more egalitarian concerns than in the intrapersonal cases of sub-optimal treatment. However, in the case of IVF, it is already the case that wealthier patients are able to access private fertility treatment that is denied to less well off patients. Given that co-payments would reduce the cost burden to patients, they would be preferable (from the point of view of equality) to the status quo.^7^

Further potential implications of cost-equivalence for IVF policy are explored in Table 5.

**Table 5:** Possible implications of cost-equivalence for IVF policy

		Pure cost-equivalence	Cost-effectiveness equivalence (or CETE)
1.	Equal access to IVF (regardless of age)	**✓**	
2.	Prognosis-adjustment. The quantity of publicly funded IVF could be linked more directly to the probability of live birth, and take into account a wider range of factors predicting probability of live birth		**✓**
3.	Co-payment. Patients would pay a variable co-payment to reflect the chance of live birth. Those with a low chance of live birth would pay a larger proportion of the cost of providing IVF		**✓**
4.	Discounted IVF. If cheaper forms of IVF become available, women with lower chance of live birth would be able to access CEE equivalent IVF by using cheaper techniques (e.g. [4])		**✓**
5.	Permit publicly funded IVF using donor eggs for older women*	**✓**	**✓**

* The chance of live birth using donor eggs appears to be related to donor age, not maternal age [30, 42]. If the justification for denying IVF to older women is on the basis of reduced chance of live birth, it would be potentially cost-equivalent to provide access to IVF using donor eggs (once the cost of oocyte donation is factored in)

### Supra-optimal Treatment

We have focused in this paper on patient requests for sub-optimal treatments. However, a more common dilemma may arise where patients request treatment that is potentially *more* effective than the current default treatment, yet is not available in the PHS. More effective therapies might not be provided because they have not yet been adequately evaluated. There may be insufficient evidence for the PHS to be sure that they are optimal (they would thus fit within our definition of *sub*optimal treatment, Table 1). Alternatively, there may be clear evidence of benefit, yet the cost of the treatments are such that they exceed the ICER threshold, and thus are judged to be unaffordable in the PHS.

Consider the following:

*Jason is a 50* *year old man who has recently diagnosed hepatitis C infection, genotype 2. He has been researching treatments for this infection, and has read national guidance recommending treatment with a new expensive anti*-*viral drug (sofosbuvir) for patients like him who do not have liver disease* [1]*. This treatment would give him the best chance of cure of his hepatitis, and has lower side effects compared with the previous standard of care (interferon)* [26]*. However, he does not have health insurance, and his PHS (Medicaid) will only fund the more expensive treatment for patients who already have liver disease* [2]*. He asks his physician to prescribe Sofosbuvir.*

The above analysis of CE suggests a number of principles that might be applied to requests for supra-optimal treatment like Jason’s.

Where patients are requesting reasonable supra-optimal treatment as a *substitute* to existing therapy, it would be fair to provide the treatment if it were cost-equivalent. That could apply either to therapies with little evidence to support them, or to more expensive therapies. On the account of reasonableness given above, there would need to be some scientific evidence of benefit (relative to no treatment), and sufficient evidence of effect and cost to assess cost-equivalence. Thus, this would apply to some novel therapies, but exclude experimental treatment with little or no published experience. Cost equivalence (either CE/CEE or CETE) could be achieved through reduction in dosage/duration, through negotiation or through co-payment. In Jason’s case, if he were to decline interferon treatment, he would be required to pay a co-payment for Sofosbuvir (discounted by the price of Interferon, see also Appendix 2).

In contrast, if the patient were to request the new treatment as a *supplement* to existing treatment, it would need to be incrementally cost-effective and cost-equivalence would not apply.

Again, the egalitarian objections to co-payment apply more forcefully to supra-optimal treatment than to sub-optimal treatment. However, as noted above in the context of IVF, given that such treatments are already available (in many cases) for patients who are willing to pay for them, cost-equivalence and co-payment potentially reduce inequality relative to the status quo by increasing accessibility to patients on low incomes.

## Conclusions

There are good reasons for Publicly-funded Healthcare Systems and health professionals to seek out and to provide the best available, affordable medical treatment to patients. However, some patients request treatment that might be judged sub-optimal from a medical point of view. We have argued in favour of supporting patients’ access to desired suboptimal treatments. Determination that treatment is optimal involves evaluating different outcomes, and potentially trading off different values. Value pluralism implies that there may be a range of different reasonable answers. We should respect and accommodate patients’ autonomous wishes and value judgments as long as their decisions do not cause harm to others. Offering a range of treatments allows patients to match therapy to their values, encouraging patients to participate in and take responsibility for their treatment choices.

We have proposed the concept of cost-equivalence as a means of defining the boundaries of permissible value pluralism within PHS. We distinguished between pure cost-equivalence, cost-effectiveness equivalence and cost-effectiveness threshold equivalence. Pure cost-equivalence is attractively simple to apply, and does not involve any evaluation of the effect of treatment. In some situations it would be Pareto-superior. Cost-Effectiveness Equivalence and Cost-Effectiveness-Threshold Equivalence provide more restrictive boundaries on access to sub-optimal treatments, but ensure that PHS achieve a reasonable health benefit overall and provide a simple way to assess the reasonableness of substitute treatments. In situations where the PHS is prepared to absorb the costs of refusing treatment, Refusal Cost-equivalence may be a useful additional principle.

Cost-equivalence could be used in a simple binary form, to adjudge the permissibility of providing treatment substitutes. It might also allow more expensive (or less effective treatments) to be provided for a shorter duration or in a smaller quantity. Cost-equivalence could also be achieved for sub-optimal treatments through price reduction (via negotiation or co-payment).

We have identified and responded to a number of potential counter-arguments to cost-equivalence. We suggest that none of them provide convincing reasons to reject our proposal. Finally, we have explored the potential use of cost-equivalence in interpersonal allocation, and in deciding about supra-optimal treatment.

In our diverse, multicultural societies, it is simply not credible that there could be a single best answer to the question of which treatment a patient should receive. At the same time, there is a need for PHS to rationally appraise different treatments, and to consistently and fairly allocate them, given finite resources. It is appropriate for PHS to identify, recommend and provide treatments that appear to offer the greatest achievable benefit. However, there is also a need to respect and support patients who make a different determination. Cost-equivalence provides a sound, fair, and rational way of doing that.

## Appendix 1

**Table 6:** Arguments and counterarguments around co-payments for cost-equivalence

Arguments against cost-equivalence co-payment	Counter-arguments (in favour of cost-equivalence co-payment)
Egalitarian Co-payments are unfair. They mean that wealthier patients are able to access treatments that less-well-off patients cannot afford [5]. Public health care should provide the same treatment to all	Inequality already exists. Patients can access treatments in the private system (if they can afford it). Co-payments reduce inequality by reducing the cost burden of such choices. Levelling down equality benefits no patients [10], but restricts choices for some
Cost-burden It is objectionable that patients might end up accruing very large medical bills in order to access treatment	Patients would not need to pay anything for the most effective available treatment. Some patients already accrue large bills (for private treatment or complementary medicine) Co-payments would reduce the bills for patients who would choose suboptimal treatments
Market effects PHS that apply strict cost-thresholds to medicines are able to negotiate with pharmaceutical companies to reduce their prices (below the ICER threshold). Co-payments would reduce the incentive for companies to drop prices, and potentially deprive other patients of treatment [21]	The number of patients choosing suboptimal treatments is likely to be low (compared to the size of the PHS)—therefore having little impact on market negotiations of the PHS [46]. On the other hand, if there were a large number of patients choosing co-payments for desired (but suboptimal) treatment, this would suggest that (a) many individuals judged the level of copayment acceptable, and (b) the PHS should reconsider its decision not to provide it
Slippery Slope Permitting co-payments (for sub-optimal treatment) would lead to wider use of co-payments within the PHS, and to progressive reduction in the funding and effectiveness of the PHS	Co-payments already exist in many PHS for some elements of healthcare (e.g. in the UK for dentistry/opticians). They have not led to a progressive decline in PHS. Co-payments for optimal treatment can be distinguished from co-payment for sub-optimal treatment. Permitting one, does not necessarily mean permitting the other

## Appendix 2

One way that Sofosbuvir could be cost equivalent would be if future costs of liver transplantation were factored into the cost of Interferon. For example, some models of Hepatitis C treatment suggest that routine funding of Sofosbuvir would avert almost 6000 cases of hepatocellular carcinoma and 121 liver transplants for every 100,000 50-year old patients treated [8]. This might make Sofosbuvir overall a cost-effective strategy. Alternatively, Jason might choose to waive his future access to transplantation in order to access the more expensive drug now. However, it is not clear that would be compatible with cost-equivalence as described here. It would also raise the possibility of him later changing his mind.

Representative costs [26]:

Interferon/Ribavirin treatment: $24,300

Sofosbuvir/Ribavirin treatment: $91,500

Liver transplant: $228,000 (in first year)

Sofosbuvir/Ribavirin is less expensive than Interferon/Ribavirin plus liver transplantation.

However, the probability of requiring a liver transplant for a patient with hepatitis C receiving Interferon is low (approximately 0.1%) [8]. If future treatments (refused) are discounted by the probability of them being required, the per patient cost of Interferon plus transplant is $24,528.

## Footnotes

1 In children, there is an additional complexity, since their own wishes may be unknown, and there are constraints to parents’ freedom to make medical decisions [47].

2 Optimal treatment is here defined by its effectiveness, but constrained by consideration of affordability (cost-effectiveness) and accessibility.

3 A Pareto Superior option is better for at least one individual, and not worse for anyone. Assuming that the substitute treatment is preferred by the patient, and does not frustrate the desires of any other patients for treatment, cost-equivalent substitute treatments are a Pareto improvement. (They may be Pareto inferior in terms of health).

4 There is some evidence to suggest that patients who refuse transfusion actually have better outcomes following surgery, and may be associated with lower costs[16, 33].

5 Note that in assessing whether suboptimal treatments fall within the incremental cost-effectiveness threshold, these treatments should be compared with no treatment or standard treatment, *not* with the optimal treatment. Since these treatments are (by definition) suboptimal, they will yield a negative value for incremental cost-effectiveness in comparison with the optimal treatment.

6 Manufacturers might agree, for example, to provide discounted treatment on compassionate grounds to patients in worse prognostic groups.

7 One further possible form of price reduction that would not raise the same egalitarian concerns is third-party co-payment. For example, a charity might elect to subsidise treatment for patients in worse prognostic groups—providing the co-payment to make it possible for them to access treatment. In such a situation, there would be no difference in access to treatment on the basis of financial resources.
